# Efficacy and safety of tripterygium wilfordii multiglycosides in idiopathic membranous nephropathy: Protocol for an open-label randomized controlled clinical trial

**DOI:** 10.1097/MD.0000000000028842

**Published:** 2022-02-11

**Authors:** Chanyu Geng, Qiang Li, Lei Pu, Hongling Yang, Guisen Li, Yunlin Feng

**Affiliations:** a Nephrology Department, Sichuan Provincial People's Hospital, Chengdu, China; b Medical School of University of Electronic Science and Technology of China, Chengdu, China; c The George Institute for Global Health, Faculty of Medicine, University of New South Wales, Sydney, Australia.

**Keywords:** efficacy, idiopathic membranous nephropathy, open-label, randomized controlled trial, safety, tripterygium wilfordii multiglycosides

## Abstract

**Objective:**

Tripterygium wilfordii multiglycosides has been demonstrated to be effective in reducing proteinuria and alleviate edema in patients with chronic kidney disease. We aim to evaluate its efficacy and safety in idiopathic membranous nephropathy.

**Methods and analysis:**

This is an randomized, open-labeled, controlled clinical trial. Twenty eligible patients with idiopathic membranous nephropathy will be randomly assigned into the intervention group and the control group at a rate of 1:1. Patients in the intervention group will receive tripterygium wilfordii multiglycosides tablets (1–1.5 mg/kg body weight/d, orally) in addition to the original treatment of angiotensin converting enzyme inhibitor/angiotensin receptor blocker, while the control group will continue with the original treatment of angiotensin converting enzyme inhibitor/angiotensin receptor blocker. The treatment course is 6 months, and clinical variables of patients will be measured at baseline and each monthly follow-up. The primary efficacy outcome measure is absolute decrease in urinary protein quantity after 6 months of treatment compared with baseline at randomization. The secondary efficacy outcome measures include absolute decrease in urine albumin-creatinine ratio in spot urine after 6 months of treatment compared with baseline at randomization, the percentage of patients who reached effective clinical response, and the percentage of patients who developed composite renal endpoint. Safety outcome measures include incidence of adverse events, incidence of serious adverse events, and death.

## 1 Introduction

Idiopathic membranous nephropathy (IMN) is one of the common pathological types of primary nephrotic syndrome in adults, with a peak age around 40 to 50 years old.^[1,2]^ In China, IMN accounts for 30.2% of primary glomerular disease and is the leading cause of nephrotic syndrome in adults over 40 years old.^[3]^ Immunosuppressive therapy is the current first line treatment of IMN, and its efficacy has been confirmed by a large number of high-level evidence.^[4]^ For IMN patients who have moderate to low risk for progressive renal function decline, the latest version of Kidney Disease: Improving Global Outcomes guideline of Chronic Glomerular Disease^[5]^ still recommends a “wait and see” policy in addition to treatment of angiotensin converting enzymes inhibitor (ACEi) or angiotensin receptor blockers (ARB). However, we have found a considerable number of patients with proteinuria below 3.5 g/d suffered from significant edema symptom, making the supporting treatment of full-dose ACEi/ARB alone less well accepted in this population, especially in the elderly.

Tripterygium wilfordii multiglycosides is extracted and purified from the root xylem of *Tripterygium wilfordii* Hook F (TwHF), which is a member of the Celastraceae family of perennial vine-like plants.^[6]^ It has been reported in literature and also shown by our experience that tripterygium wilfordii multiglycosides can reduce proteinuria and alleviate edema symptom of patients with chronic kidney disease,^[7–9]^ through mechanisms including but not limited to activating T cells^[10]^ and regulating nuclear factor κB pathway.^[7,10,11]^ Most experience of tripterygium wilfordii multiglycosides in IMN so far came from observational studies with limited number of subjects. There is still a lack of solid evidence to support its use in IMN patients.

This is a randomized, open-label, controlled trial to investigate the efficacy and safety of tripterygium wilfordii multiglycosides in IMN patients who would be classified as moderate to low risk according to Kidney Disease: Improving Global Outcomes guideline. The results might be used to improve our understanding of this drug in treating IMN and provide evidence for clinical practice. We hypothesized that tripterygium wilfordii multiglycosides combined with ACEi/ARB is superior to ACEi/ARB alone in treating IMN.

## 2 Methods and analysis

### 2.1 Study design

**Figure 1:**
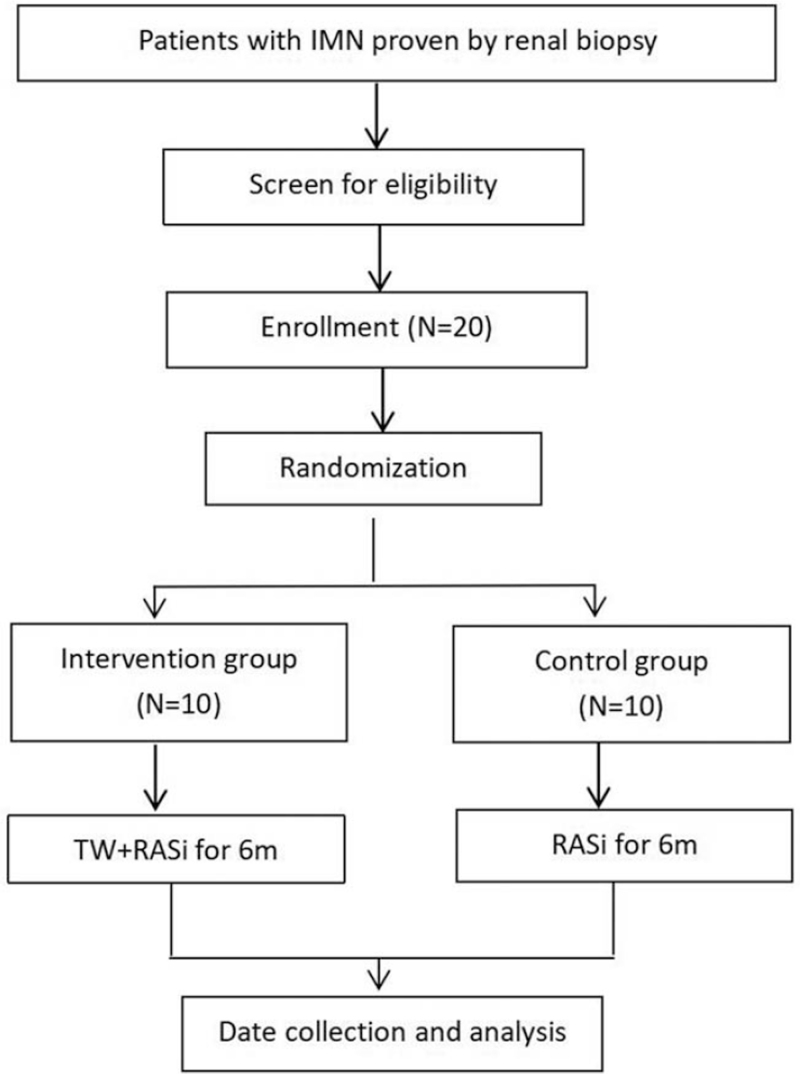
Flowchart of the trial. m = months, RASi = renin-angiotensin system inhibitors, TW = tripterygium wilfordii multiglycosides.

This is a single center, open-label randomized controlled clinical trial following superiority trial design, and is sponsored by Nephrology Department of Sichuan Provincial People's Hospital. A flowchart of the trial design is shown in Fig. 1. The study has been approved by the Ethics Committee of Sichuan Provincial people's Hospital (No. 2021.325–1), and registered on Chinese Clinical Trial Registry (Identifier# ChiCTR2100048382).

### 2.2 Patients

Patients diagnosed as IMN by renal biopsy will be included in this study. Twenty eligible IMN patients will be randomly assigned to an intervention group and a control group at a rate of 1:1, with 10 patients in each group. Patients in the intervention group will receive tripterygium wilfordii multiglycosides tablets on the basis of ACEi/ARB treatment, while patients in the control group will continue with ACEi/ARB treatment. The study duration will be 6 months, during which clinical data and samples will be collected according to a visit schedule at each follow-up.

### 2.3 Inclusion criteria

Patients should meet all following criteria to be considered eligible: has no evidence of secondary membranous nephropathy; age between 18 and 75 years old; has 24-hour albuminuria between 1.0 and 3.5 g after at least 3 months of standard dose of ACEi/ARB treatment; estimated glomerular filtration rate (eGFR) >60 mL/min/1.73 m^2^ according to Chronic Kidney Disease Epidemiology Collaboration formula using the latest serum creatinine value at screening; and agrees to voluntarily participate in the study and signs an informed consent form.

### 2.4 Exclusion criteria

Patients with any one of the following criteria will be excluded: has received glucocorticoids or other immunosuppressants in the past 6 months; has any clinical evidence of secondary membranous nephropathy (tumors, drugs, other autoimmune diseases such as systemic lupus erythematosus, etc); has any of the following comorbidities including chronic liver disease, myocardial infarction, stroke, uncontrolled hypertension and diabetes, malignant tumor; is known to allergic to tripterygium wilfordii multiglycosides or has contraindications for use; female patients who are pregnant (or intend to be pregnant within half a year), breastfeeding or unwilling to take contraceptive measures; has an expected life expectancy <6 months; is currently participating or have participated in other clinical trials within 3 months; refuses to sign an informed consent form or be unable or unwilling to comply with research protocol approved by the researcher; has any other conditions which the researchers believe makes the subject unsuitable for this study.

### 2.5 Randomization

A random number list has been generated by the first author (CYG) using SPSS version 24.0 (IBM Corporation, Chicago, Illinois, USA) using simple randomization method. Eligible patients will be randomly assigned to the interventional group or the control group at a rate of 1:1 by the number inside a concealed envelope containing random codes according to the order of enrollment. The concealed envelopes have been prepared by an irrelevant researcher.

### 2.6 Interventions

All patients will have received ACEi/ARB treatment for at least 3 months before enrollment. After randomization, patients in the interventional group will receive tripterygium wilfordii multiglycosides tablets (1–1.5 mg/kg body weight/d, orally, divided to 3 times) in addition to their ACEi/ARB treatment, and patients in the control group will proceed with their original ACEi/ARB treatment. The overall treatment duration is 6 months. During the study period, existing treatments for concomitant medical conditions are permitted and should be recorded in details.

### 2.7 Outcome measurement

#### 2.7.1 Efficacy outcomes

Primary efficacy outcome is absolute decrease in proteinuria after 6 months of treatment compared with baseline at randomization.

Secondary outcomes include: absolute decrease in urine albumin-to-creatinine ratio (ACR) after 6 months of treatment compared with baseline at randomization; the percentage of patients who have achieved complete response, defined as proteinuria less than 0.3 g/d and serum albumin ≥3.5 g/dL; the percentage of patients who have achieved partial response, defined as a decrease in proteinuria ≥50% compared with the level at randomization.

#### 2.7.2 Safety outcomes

Since the major adverse effects of tripterygium wilfordii multiglycosides are liver dysfunction and gonadal suppression,^[12,13]^ we will closely monitor manifestations of these 2 aspects in addition to routine practice on safety surveillance. Therefore, safety outcomes in this study will include but not limited to the following:

- Clinical: blood pressure, edema, anemia, menstruation status in the women;
- Blood biological tests: complete blood cell count; serum creatine, albumin, alanine aminotransferase, aspartic aminotransferase, total bilirubin, direct bilirubin, indirect bilirubin, lactate dehydrogenase.

An adverse events (AE) mean any adverse event that happens during the study duration, regardless of its relevance to the experimental intervention. Any adverse event happens will be recorded in details and followed up until resolution. Serious AE includes life-threatening conditions, hospitalization, prolonged hospitalization, transient or permanent disability, and death. Any serious AE should be reported to the sponsor within 24 hours after occurrence, recorded, and followed as AE.

### 2.8 Data collection and management

All enrolled patients will have their first visit at the time of screening, the second visit at randomization and be followed up at the end of each month afterwards (Table 1). In addition, there will be 1 follow up at 2 weeks after randomization (Visit 3), whose main purpose is to closely monitor potential adverse effect of tripterygium wilfordii multiglycosides. The overall duration of treatment will be 6 months. At each follow-up as shown in Table 1, the medical history will be enquired, and blood and urine tests will be carried out in the central lab in Sichuan Provincial People's Hospital with standardized methods.

**Table 1:** Visit schedule of this study.

Research program	Screening	Randomization				Clinic visit			
Visit number	1	2	3	4	5	6	7	8	9
Visit day	−14 (±7)	0	14 (±3)	30 (±3)	60 (±3)	90 (±3)	120 (±3)	150 (±3)	180 (±3)
Informed consent	√								
Inclusion/Exclusive criteria	√	√							
Random assignment		√							
Demographics	√								
Medical history	√	√	√	√	√	√	√	√	√
Physical examination	√	√	√	√	√	√	√	√	√
Vital signs	√	√	√	√	√	√	√	√	√
Blood lab tests		√	√	√	√	√	√	√	√
Urine lab tests		√	√	√	√	√	√	√	√
Collect blood and urine samples		√			√		√		√
Pregnancy test	√	√							
Combined medication		√	√	√	√	√	√	√	√
Adverse events		√	√	√	√	√	√	√	√
Drug distribution		√	√		√	√	√	√	
Drug compliance reminder		√	√		√	√	√	√	

In this study, the paper version of CRF table will be used for data collection, RedCap online database will be used to manage data, double input comparison method will be used for data entry and verification, and corresponding data verification and cleaning will be carried out. During the study period, the principle researchers will check the trial data monthly to ensure internal consistency and data integrity.

### 2.9 Withdraw criteria

Patients with any of the following conditions will be withdrawn from this study: being found having any of the exclusive criteria after enrollment; takes glucosteroids or other immunosuppressants during the study; has intolerable side effects of drugs; has any serious adverse event that makes subjects unsuitable for continuation, such as severe infection, uncontrollable hyperglycemia, and/or hypertension; the patient asks to withdraw; other situations which the researchers believe makes the patient not appropriate to continue.

### 2.10 Sample size

In a preliminary screening from January 2010 to December 2017 in our center's Renal Treatment System, a total of 87 IMN patients were identified. The average amount of proteinuria in this population was 2.89 ± 0.62 g/d at the time of diagnosis. According to the clinical data of our center, combined with the published data on proteinuria in IMN patients, we expect that the experimental treatment is superior to the control when mean decrease in proteinuria in the intervention group is 2.0 g/d higher than that in the control group. Therefore, the sample size calculated with type I error of 0.05 (bilateral) and a randomization ratio of 1:1 to provide a power of 80% to detect an effect size of 2 g/d is 9 per group. The total number increased to 20 considering a loss of follow-up rate of 10%. Minimum loss to follow-up is expected given such small sample size and intensive clinic visits during follow-up. Therefore, 20 subjects will be included in this study.

### 2.11 Data analysis

Continuous data will be described by mean ± standard deviation or median (interquartile range). Classified data will be described by the number and percentages. Chi-square test or Fisher exact test (if expected number in 1 cell is below 5) for categorical variables and Student *t* test or Wilcoxon rank sum test for continuous variables will be applied to detect the differences between the intervention and the control groups. Due to the potential spontaneous remission in IMN patients, analysis of covariance will be conducted using proteinuria at baseline as a covariant to further evaluate the difference between these 2 groups. A 2-tailed *P* < .05 will be considered to be significant. Statistical analysis will be performed using SPSS version 24.0 (IBM Corporation, Armonk, NY) and RStudio for Windows, version 4.0.3 (RStudio, Inc., Boston, MA, USA) and supervised by a skilled statistician (QL).

## 3 Discussion

IMN is one of the most common primary glomerular diseases in China, especially in the elderly, and its prevalence is rising over recent years.^[14]^ Patients who are classified as low risk of renal function decline based on Kidney Disease: Improving Global Outcomes guidelines often present with obvious lower extremities edema, which make the supportive treatment of ACEi/ARB alone less well accepted.

Tripterygium wilfordii multiglycosides has been used to treat rheumatic disease in China for many years. Evidence from a number of observational studies indicated this drug can reduce proteinuria and alleviate edema of patients with chronic kidney diseases including IMN.^[12]^ These findings are consistent with our own clinical experience. Previous studies have shown that tripterygium wilfordii multiglycosides can significantly reduce the production of inflammatory cytokines such as tumor necrosis factor α, Interleukin-1β, and monocyte chemoattractant protein-1 and ameliorated oxidative stress.^[10]^ It can attenuate inflammatory response by down-regulation of nuclear factor κB signaling pathway.^[10,11]^ In addition, tripterygium wilfordii multiglycosides can also promote the structural repair of podocyte hiatus membrane protein.^[7]^ These mechanisms might explain the reduction of proteinuria observed. However, there is still a lack of high-level evidence for using this drug to treat IMN. Therefore, we designed this randomized, open-label, controlled trial to investigate its efficacy and safety in treating this disease.

Tripterygium wilfordii multiglycosides has known side effects, of which hepatotoxicity and menstrual disorder are the top 2. Excessive dose and prolonged duration are the main reasons to cause adverse effects.^[13]^ The reported incidence of reproductive toxicity among tripterygium wilfordii multiglycosides users thus far was between 3.4% and 7.9%.^[14]^ In our study, we will strictly calculate and control the dose and treatment duration of each subject to avoid drug overdose and accumulation. Meanwhile, we will closely monitor adverse effects in monthly follow up visit, and we also add 1 follow-up (Visit 3) to monitor potential adverse effects of tripterygium wilfordii multiglycosides. If any signs intolerable side effects relevant to the experimental drug occurs, we will withdraw the subject immediately, take appropriate measures, and follow up until the side effect resolves.

It should be noted the calculated sample size is rather small, only 10 for each group considering a loss of follow-up rate of 10%. Thus, strict adherence to study protocol is of great importance to the success of this trial and every effort to keep the patient being followed up as planned. Our department is a core member of China's Chronic Kidney Disease Management Center and has an sophisticate electronic system which combines the advantages of on-site and remote health management to track our patients. The system sends mobile messages to patients who are close to their appointed visits to remind them to show up at clinic. We also have a designated nurse to manage patients’ follow up. All these measures aim to keep patients in regular follow up. In addition, 4 authors of this work are all senior nephrologists. With the help of our CKDMC's management, we will try every effort to achieve a maximum compliance and minimum loss of follow up rate in this study.

In summary, this study aims to provide scientific evidence for tripterygium wilfordii multiglycosides in treating IMN through a randomized, open-labeled, controlled clinical trial. The results may provide evidence to support treatment of tripterygium wilfordii multiglycosides in IMN, and hopefully have important significance for clinicians who are to make therapeutic decisions.

### 3.1 Ethics and dissemination

The study received approval from the Ethics Committee of Sichuan Provincial people's Hospital (No. 2021.325–1). The study will be conducted following Ethical Principles of Helsinki Declaration and relevant local clinical research norms and regulations. Written informed consent will be acquired from every participant before study-related procedure. A verification of the consents will be carried out after each inclusion, followed by an auditing of the files at regular time intervals. The participants’ personal data will be kept confidential throughout the study and only available to the research team. Any change to the protocol should be approved by the Ethics Committee before it can be implemented and will be applied to the clinical registry for protocol changes. Trial results will be communicated to participants, healthcare professionals, the public, and other relevant groups via publication.

## Acknowledgment

The authors thank Dr Daqing Hong for his assist in preparing concealed envelopes.

## Author contributions

Yunlin Feng conceived the study and wrote the manuscript. Chanyu Geng wrote the manuscript and led the registration. Qiang Li, Lei Pu, Hongling Yang, and Guisen Li contributed to the study design and critically reviewed the manuscript. All authors read and approved the final manuscript.

**Conceptualization:** Guisen Li, Yunlin Feng.

**Data curation:** Chanyu Geng, Qiang Li, Lei Pu, Hongling Yang, Yunlin Feng.

**Formal analysis:** Qiang Li, Guisen Li, Yunlin Feng.

**Funding acquisition:** Yunlin Feng.

**Supervision:** Guisen Li.

**Writing – original draft:** Chanyu Geng.

**Writing – review & editing:** Qiang Li, Lei Pu, Hongling Yang, Guisen Li, Yunlin Feng.
